# Multiple metals influence distinct properties of the Arabidopsis circadian clock

**DOI:** 10.1371/journal.pone.0258374

**Published:** 2022-04-05

**Authors:** Jessica K. Hargreaves, Rachael J. Oakenfull, Amanda M. Davis, Freya Pullen, Marina I. Knight, Jon W. Pitchford, Seth J. Davis

**Affiliations:** 1 Department of Mathematics, University of York, York, United Kingdom; 2 Department of Biology, University of York, York, United Kingdom; 3 State Key Laboratory of Crop Stress Biology, School of Life Sciences, Henan University, Kaifeng, China; Karlsruhe Institute of Technology, GERMANY

## Abstract

Circadian rhythms coordinate endogenous events with external signals, and are essential to biological function. When environmental contaminants affect these rhythms, the organism may experience fitness consequences such as reduced growth or increased susceptibility to pathogens. In their natural environment plants may be exposed to a wide range of industrial and agricultural soil pollutants. Here, we investigate how the addition of various metal salts to the root-interaction environment can impact rhythms, measured via the promoter:luciferase system. The consequences of these environmental changes were found to be varied and complex. Therefore, in addition to traditional Fourier-based analyses, we additionally apply novel wavelet-based spectral hypothesis testing and clustering methodologies to organize and understand the data. We are able to classify broad sets of responses to these metal salts, including those that increase, and those that decrease, the period, or which induce a lack of precision or disrupt any meaningful periodicity. Our methods are general, and may be applied to discover common responses and hidden structures within a wide range of biological time series data.

## 1 Introduction

Many species exhibit changes in behaviour between day and night, with these daily rhythms (known as ‘circadian rhythms’) being the predictive result of an internal timekeeping system which responds to daily changes in the physical environment [1–3]. Like many organisms, plants sense various environmental conditions, such as light and temperature, and integrate this information with their circadian clock to measure day length and seasonal change [4]. This integration can increase fitness for organisms, as the ‘circadian clock’ enhances functions such as growth and survival by directing anticipatory changes in physiology synchronised with environmental fluctuations [5, 6].

As sessile organisms, plants depend on this synchronisation as it allows the anticipation of daily and seasonal changes and therefore stresses [7] such as temperature [8], drought [9] and pathogen response [10]. The circadian clock also allows the synchronisation of water uptake [11], photosynthesis [12] and growth [13]. In crop species, a robust circadian oscillator allows higher crop yields as the plant is maximally efficient in growth [14].

Mathematical models of the circadian clock have been developed for many organisms in order to facilitate understanding of the circadian clock mechanism [15]. In the laboratory model plant species, *Arabidopsis thaliana* (henceforth ‘Arabidopsis’ or *‘A. thaliana’*), various models for the central oscillator have been developed, from a simple positive–negative feedback model [16] to more complex interconnected feedback loops [17]. The mechanistic basis of the Arabidopsis circadian clock is well understood [18]. However, it is less clear how the central oscillator is modulated by external cues such as nutritional status or soil composition [19]. Quantifying and characterising the effects of these factors is the focus of our work here.

### 1.1 Statistical developments

One of the keys steps in identifying the molecular components of the circadian clock is to examine the rhythmicity (or arhythmicity) of experimental time series data [15]. Traditionally, the analysis of (rhythmic) circadian data has centred around the investigation of how certain circadian clock parameters (periodicity, phase, amplitude and clock precision) are affected (S1 Fig provides a brief introduction into the nature of these parameters [4, 20, 21]).

Period estimation has been central to the analysis of circadian data and a range of estimation and analysis techniques (e.g., Enright and Lomb–Scargle periodograms [22, 23], mFourfit [24], MESA [25] and Spectrum Resampling [26]) are available. The current industry standard is to implement Fourier analysis techniques [15, 26] such as FFT–NLLS (Fast Fourier Transform Non-Linear Least Squares [27]) via software such as BRASS (Biological Rhythm Analysis Software System [24]) or BioDare [28]. The practitioner estimates the period of the control and treatment groups respectively, and then tests for statistically significant differences (see for example [26, 29]). When certain assumptions are valid, such as the data being underpinned by a trigonometric function with constant period and amplitude, this is an effective analysis method [15].

When the data depart from such assumptions through, for example, displaying a changing period and/or amplitude (also known as a loss of ‘clock precision’), the application of such period estimation methodology may lead to misleading conclusions [30]. Wavelets have been recognised as ideally suited to identifying localised time and scale features in circadian rhythms [20, 31, 32], with time-scale spectral patterns known to be indicative of the organism response to external stimuli [15, 33].

This motivates our choice to adopt, alongside traditional Fourier–based methods, the wavelet–based spectral testing and clustering methods of [20, 30] in order to analyse our experimental circadian dataset. For nonstationary circadian signals, these wavelet–based methods allow formal comparisons in the time-scale spectral domain and are ideally suited for an analysis across a diverse range of environmental conditions, where biologically important effects are observed which lie beyond the scope of simple changes in period or phase.

### 1.2 Environmental context

The plant circadian clock enhances growth and survival by directing anticipatory changes in physiology synchronised with environmental fluctuations [5, 6]. In recent years, there has been an increasing awareness of the ionic control of clock performance [34]. Reports investigating the roles of calcium, copper, iron, zinc and magnesium ions on circadian performance illustrate the critical nutritional role of several metals and the homeostatic relations between them [35–37]. Indeed the increased concentration of several of these metals causes alterations in signalling, and it is known that the uptake and transport of all of these ions are themselves subject to circadian regulation [29, 38–40]. Another area of concern regards the contamination of soils [41]. For example, high concentrations of heavy metals in agricultural soils can occur naturally or via the application of metal–contaminated sewage sludge, fertilisers or animal manures [14, 42]. One report has shown that the presence of the heavy metal cadmium alters clock activity [43]. In addition, increases in sodium are known to perturb normal clock function [44].

Soil quality includes an important balance of numerous ions and organic compounds. Contamination of soil can be defined in broad terms as any alteration in the natural soil environment, with common causes consisting of industrial activity, the application of agricultural chemicals and the improper disposal of waste [41, 45–48]. As a result of soil pollution, plant growth conditions may change in various ways through exposure to essential nutrients at toxic levels, or to non–essential elements not usually encountered in the natural environment [49]. Soil contaminants can also significantly impact ecosystems. In particular, changes in soil chemistry which affect the numbers and fitness levels of plants, may have major consequences throughout the food chain as species respond to changes in the food supply [14, 49–51].

These are compelling arguments for addressing the current scientific understanding of the effects of soil ionosphere on the plant circadian clock (see also [42, 44]). As the addition of several cations to the root systems perturbs shoot rhythms in reporter assays, here we examine these processes in a more holistic way, by adding a suite of salts to standard growth media and assessing the consequence on luminescence rhythms in individual plants measured as time series over days of growth. We infer numerous cation and several anion effects on clock activity. Considering the periodic table and all of the potential ions a plant could be exposed to (via natural, fertilized or waste-contaminated regimes), this experimental approach, coupled with comprehensive state-of-the-art statistical analyses, means that the experimental data reported here comprise a near-comprehensive set of results on the ways that soil pollution can impact the plant circadian clock.

### 1.3 Aims and structure of the paper

In this article, we present a holistic investigation of the effects of increasing ion levels on clock rhythms. Specifically, we investigate the impact of exposure to a comprehensive range of chemicals on the Arabidopsis clock.

This article is organised as follows. Section 2.1 outlines the experimental details that led to the datasets analysed in this paper. Section 2.2 presents the Fourier and novel wavelet-based statistical methodologies we employ. Section 3 connects the results obtained using the FFT–NLLS-based methods to those obtained from the wavelet spectral testing and clustering methodology. Section 4 briefly discusses the value of this comprehensive analysis, both in terms of its relevance to plant growth and environmental pollution, and of its general value for the analysis of high-dimensional time series datasets.

## 2 Materials and methods

### 2.1 Experimental procedures

In this section, we briefly outline the experimental procedures that gave rise to the rich circadian time–series data that we subsequently analyse in Section 3 (full details are given in Sections 2.1.1–2.1.5).

We investigated whether the 49 elements detailed in Tables 1–3 impact the Arabidopsis circadian clock, when applied to basal media typical of numerous circadian studies. The 11 elements listed in Table 1 comprise of chemicals at concentrations indicative of pollution, as defined by the UK Department for Environment, Food and Rural Affairs (DEFRA) in Part 2A of [52]. Driven by practical relevance [29, 38–40], we extend the analysis to encompass a further list of chemicals (Tables 2 and 3).

**Table 1:** Chemical treatments: Elements described in the DEFRA/YARA/Lancrop guidelines [52].

AN	Element	Salt	Conc.	Period Difference	RAE	Rejections FT (FDR)
9	Fluorine (F)	NaF	26mM (Max)	3.27	0.56†	**501 (56%)**
9	Fluorine (F)	NaF	13mM (Half)	0.39	0.18	**15 (2%)**
24	Chromium (Cr)	KCr(SO_4_)_2_	7mM (Max)	NA	NA	**594 (66%)**
24	Chromium (Cr)	KCr(SO_4_)_2_	3.5mM (Half)	-1.18	0.60†	**544 (61%)**
28	Nickel (Ni)	NiCl_2_	10mM (Max)	0.96	0.51†	**534 (60%)**
28	Nickel (Ni)	NiCl_2_	500*μ*M (Half)	1.41	0.53†	**498 (56%)**
29	Copper (Cu) ‡	CuSO_4_	1.6mM (Max)	2.82	0.92†	**475 (53%)**
29	Copper (Cu)	CuSO_4_	800*μ*M (Half)	-2.66	0.88†	**442 (49%)**
30	Zinc (Zn) ‡	ZnSO_4_	3mM (Max)	**0.56** *	0.17	**90 (10%)**
30	Zinc (Zn)	ZnSO_4_	1.5mM (Half)	0.15	0.14	3 (0%)
33	Arsenic (As) ‡	KAsO_4_	670*μ*M (Max)	1.94	0.42	**458 (51%)**
33	Arsenic (As) ‡	KAsO_4_	335*μ*M (Half)	1.59	0.31	**123 (14%)**
34	Selenium (Se) ‡	Na_2_SeO_4_	40*μ*M (Max)	**3.83** *	0.21	**196 (22%)**
34	Selenium (Se) ‡	Na_2_SeO_4_	20*μ*M (Half)	**2.48** *	0.20	**198 (22%)**
42	Molybdenum (Mo) ‡	Na_2_MoO_4_	4mM (Max)	-3.18*	0.65†	**346 (39%)**
42	Molybdenum (Mo) ‡	Na_2_MoO_4_	2mM (Half)	**-3.99** *	0.32	**284 (32%)**
48	Cadmium (Cd)	CdCl_2_	26*μ*M (Max)	0.17	0.22	3 (0%)
48	Cadmium (Cd) ‡	CdCl_2_	13*μ*M (Half)	0.38	0.22	1 (0%)
48	Cadmium (Cd)	CdSO_4_	26*μ*M (Max)	-0.32	0.20	1 (0%)
48	Cadmium (Cd)	CdSO_4_	13*μ*M (Half)	0.28	0.21	1 (0%)
80	Mercury (Hg) ‡	HgCl_2_	5*μ*M (Max)	-0.06	0.15	1 (0%)
80	Mercury (Hg)	HgCl_2_	2.5*μ*M (Half)	0.13	0.18	1 (0%)
82	Lead (Pb) ‡	Pb(NO_3_)_2_	1.4mM (Max)	-0.62	0.32	**133 (15%)**
82	Lead (Pb) ‡	Pb(NO_3_)_2_	700*μ*M (Half)	**-0.70** *	0.20	1 (0%)

The salts and concentrations (denoted “Conc.”) used in this experiment, where “Element” represents the element under investigation within the chemical compound (corresponding to the periodic table representation used in Fig 3) and “AN” represents the associated atomic number. For each chemical, the number of rejections (as a percentage in brackets) for the LSW–FT with FDR (at the 5% significance level) is reported along with the estimated mean difference in period and mean RAE (using FFT–NLLS), with:

* indicating a significant change in period from the respective control group and

^†^ denoting an RAE value above the 0.5 threshold.

**Bold font** indicates a usable result (i.e. a statistically significant result which also follows the specific method’s usability criteria). The symbol ‡ indicates that the chemical was used in the cluster analysis in Section 3.2.1.

**Table 2:** Extension chemicals: Part 1 (atomic numbers 3–27).

AN	Element	Salt	Conc.	Period Difference	RAE	Rejections FT (FDR)
3	Lithium (Li)	LiCl_2_	20mM	**4.54** *	0.19	**280 (31%)**
3	Lithium (Li)	Li_2_SO_4_	15mM	6.76	0.43	**455 (51%)**
5	Boron (B) ‡	Na_2_B_4_O_7_	3mM	**-1.68** *	0.10	**34 (4%)**
11	Sodium (Na)	NaCl	2mM	-0.21	0.17	1 (0%)
11	Sodium (Na)	NaBr	100mM	**1.33** *	0.21	**114 (13%)**
11	Sodium (Na)	NaI	100mM	0.32	0.34	**545 (61%)**
12	Magnesium (Mg)	MgCl_2_	5mM	-1.21	0.23	**38 (4%)**
12	Magnesium (Mg) ‡	C_4_H_6_O_4_Mg	5mM	**2.00** *	0.11	**512 (57%)**
12	Magnesium (Mg)	Mg(NO_3_)_2_	5mM	0.05	0.16	2 (0%)
13	Aluminium (Al)	AlCl_3_	300*μ*M	-0.45	0.19	**6 (1%)**
14	Silicon (Si)	Na_2_SiO_3_	25mM	0.56	0.23	**7 (1%)**
19	Potassium (K)	KCl	100mM	**1.55** *	0.17	**146 (16%)**
19	Potassium (K)	KBr	100mM	**1.55** *	0.18	**95 (11%)**
19	Potassium (K)	KI	100mM	**-1.42** *	0.22	**252 (28%)**
20	Calcium (Ca)	CaCl_2_	50mM	**1.77** *	0.31	**9 (1%)**
20	Calcium (Ca)	Ca(NO_3_)_2_	1mM	0.08	0.13	2 (0%)
21	Scandium (Sc)	Sc(SO_3_CF_3_)_3_	100*μ*M	0.20	0.19	1 (0%)
21	Scandium (Sc)	ScF_3_	300*μ*M	-0.58	0.18	1 (0%)
23	Vanadium (V)	H_3_NO_3_V	25*μ*M	-0.41	0.25	**4 (1%)**
25	Manganese (Mn)	MnCl_2_	1mM	**0.87** *	0.15	**19 (2%)**
25	Manganese (Mn)	MnSO_4_	200*μ*M	0.48	0.13	1 (0%)
26	Iron (Fe)	FeCl_3_	300*μ*M	**-1.27** *	0.16	**16 (2%)**
27	Cobalt (Co)	CoCl_2_	250*μ*M	**1.70** *	0.25	**133 (35%)**
27	Cobalt (Co)	CoSO_4_	250*μ*M	**1.82** *	0.23	**158 (41%)**

The salts and concentrations (denoted “Conc.”) used in this experiment, where “Element” represents the element under investigation within the chemical compound (corresponding to the periodic table representation used in Fig 3) and “AN” represents the associated atomic number. For each chemical, the number of rejections (as a percentage in brackets) for the LSW–FT with FDR (at the 5% significance level) is reported along with the estimated mean difference in period and mean RAE (using FFT–NLLS), with:

* indicating a significant change in period from the respective control group and

^†^ denoting an RAE value above the 0.5 threshold.

**Bold font** indicates a usable result (i.e. a statistically significant result which also follows the specific method’s usability criteria).

^‡^ indicates that the results for this chemical are discussed in detail in Section 3.

**Table 3:** Extension chemicals: Part 2 (atomic numbers 37–83).

AN	Element	Salt	Conc.	Period Difference	RAE	Rejections FT (FDR)
37	Rubidium (Rb)	RbCl	200*μ*M	0.38	0.20	2 (0%)
38	Strontium (Sr)	SrCl_2_	30mM	**1.42** *	0.25	**189 (21%)**
39	Yttrium (Y)	YCl_3_	3mM	**-3.18** *	0.49	**418 (47%)**
41	Niobium (Nb)	NbCl_5_	500*μ*M	-0.39	0.22	**2 (1%)**
44	Ruthenium (Ru)	RuCl_3_	2mM	**0.64** *	0.13	1 (0%)
47	Silver (Ag)	AgNO_3_	200*μ*M	-0.46	0.28	**50 (6%)**
50	Tin (Sn)	SnCl_2_	1.5mM	**-1.81** *	0.32	**43 (11%)**
55	Caesium (Cs)	CsCl	200*μ*M	0.27	0.18	4 (0%)
57	Lanthanum (La) ‡	LaCl_3_	5mM	-3.33*	0.53†	**420 (47%)**
58	Cerium (Ce)	CeCl_3_	3mM	**-2.83** *	0.29	**630 (70%)**
58	Cerium (Ce)	(NH_4_)_2_Ce(NO_3_)_6_	150*μ*M	-1.40	0.47	**281 (31%)**
59	Praseodymium (Pr)	PrCl_3_	2mM	**-2.53** *	0.23	**625 (70%)**
60	Neodymium (Nd)	NdCl_3_	1.5mM	0.62	0.23	**40 (4%)**
63	Europium (Eu)	EuCl_3_	5mM	**-2.02** *	0.38	**490 (55%)**
64	Gadolinium (Gd)	(CF_3_SO_3_)_3_Gd	500*μ*M	0.57	0.27	**27 (3%)**
64	Gadolinium (Gd)	GdCl_3_	600*μ*M	0.03	0.19	1 (0%)
65	Terbium (Tb)	TbCl_3_	1.5mM	**-2.60** *	0.43	**541 (60%)**
66	Dysprosium (Dy)	DyCl_3_	3mM	-1.56*	0.55†	**501 (56%)**
66	Dysprosium (Dy)	DyF_3_	100*μ*M	0.66	0.29	**2 (1%)**
67	Holmium (Ho)	HoCl_3_	1mM	**-2.51** *	0.44	**447 (50%)**
68	Erbium (Er)	ErCl_3_	1mM	**-1.92** *	0.27	**617 (69%)**
69	Thulium (Tm)	TmCl_3_	1mM	-2.48*	0.54†	**412 (46%)**
70	Ytterbium (Yb)	YbCl_3_	1mM	-2.64*	0.58†	**592 (66%)**
71	Lutetium (Lu)‡	LuCl_3_	1mM	0.92	0.25	119 (13%)
74	Tungsten (W)	Na_2_WO_4_	20g/L	1.61	0.25	**119 (31%)**
78	Platinum (Pt)	K_2_PtCl_4_	200*μ*M	**-3.62** *	0.22	**409 (46%)**
79	Gold (Au)	KAuCl_4_	50*μ*M	0.10	0.17	3 (0%)
83	Bismuth (Bi)	BiCl_3_	2mM	**-1.10** *	0.23	**179 (20%)**

The salts and concentrations (denoted “Conc.”) used in this experiment, where “Element” represents the element under investigation within the chemical compound (corresponding to the periodic table representation used in Fig 3) and “AN” represents the associated atomic number. For each chemical, the number of rejections (as a percentage in brackets) for the LSW–FT with FDR (at the 5% significance level) is reported along with the estimated mean difference in period and mean RAE (using FFT–NLLS), with:

* indicating a significant change in period from the respective control group and

^†^ denoting an RAE value above the 0.5 threshold.

**Bold font** indicates a usable result (i.e. a statistically significant result which also follows the specific method’s usability criteria).

^‡^ indicates that the results for this chemical are discussed in detail in Section 3.

Respective salts containing the elements of interest were exogenously added in 99 separate experiments to ascertain whether, and how, these elements influence circadian rhythms, as assayed via the promoter *luciferase* (LUC) system [53] (see Section 2.1.5 for further details). Seedlings harbouring a promotor:LUC marker were entrained under 12 hours light/12 hours dark cycles and then transferred into imaging plates containing either (Hoagland [54]) growth medium only (control groups), or growth medium and the test chemical (treatment groups) [4].

For the elements described in the DEFRA/YARA/Lancrop guidelines [52] (listed in Table 1) we report the results of testing at two concentrations: the maximum permissible concentration [52] and half of the maximum concentration for the pH of the media used (5.5 < 6.0), denoted “(Max)” and “(Half)”, respectively. For all other elements (Tables 2 and 3), we report the results of testing at one concetration. The applied concentration for each (see Tables 2 and 3) was defined by the maximum concentration possible before becoming toxic to the plant. (See Section 2.1.5 for further details).

**Fig 1:**
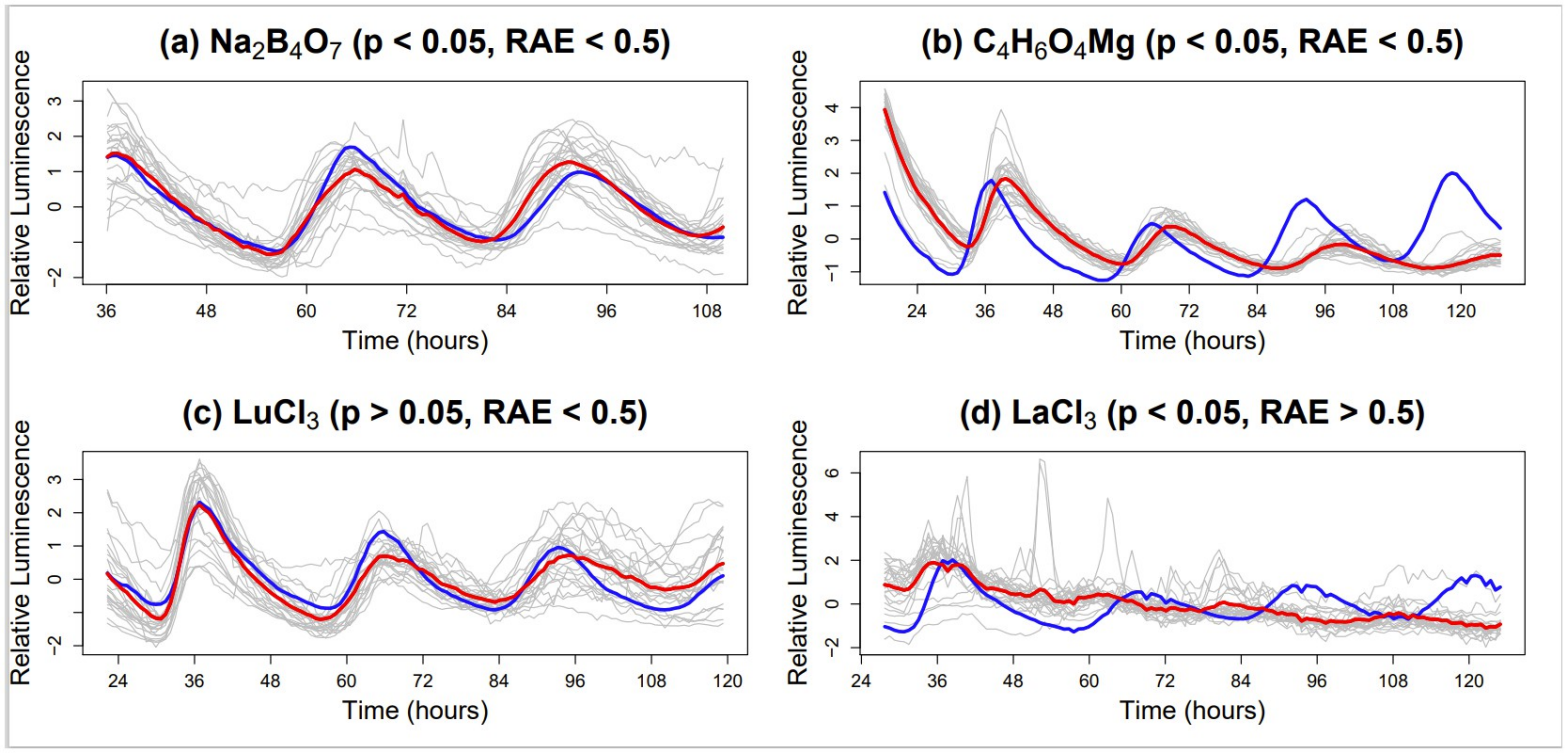
Excess external chemicals vary in their effects on plant circadian rhythms. Examples of statistically significant (a) period shortening and (b) period lengthening (FFT–NLLS, *p* < 0.05, *RAE* < 0.5) and examples (c, d) showing clear circadian disruption not captured by FFT–NLLS (*p* > 0.05 or *RAE* > 0.5). Each panel: individuals in the treatment group (grey) are shown along with the treatment group average (red) and the control group average (blue). Each time series has been standardised to have mean zero.

In what follows, “chemical treatment” denotes a specific salt tested at a certain concentration (i.e. one row in Tables 1–3). We followed the circadian rhythms of transcription rates from the well–characterised marker gene *COLD– AND CIRCADIAN–REGULATED 2* (*CCR2*), also termed *GRP7* [55]. These experiments were under free-running constant–light conditions (LL), under the standard protocol we reported [53]. Chemical effects on the CCR2-driven bioluminescence rhythms under free-running constant–light conditions are illustrated in Fig 1.

#### 2.1.1 Plant materials and chemicals

Arabidopsis thaliana lines wassilewskija (WS-2) ecotype harbouring luciferase driven by the *CCR2::LUC* promoter were obtained from [53].

#### 2.1.2 Growth media

Plants were grown on Hoagland’s media as described in [54] with the addition of 1% (w/v) sucrose, 0.5 g/litre MES (2-N-Morpholinoethane sulphonic acid) and 1.5% (w/v) phyto-agar (Duchefa), solution adjusted to Ph 5.7 with KOH.

#### 2.1.3 Growth conditions

WS-2 *CCR2:LUC* seeds surface sterilised and plated onto Hoagland’s media with 1% sucrose. Stratified for 48 hours at 4°C before moving to a growth chamber (for 6 days) with a constant temperature of 20°C and light regime of 12 hours light 12 hours dark.

#### 2.1.4 Microtitre plate assembly and Luminescence assay

Following a modified version of the method described in [21], black 96 well microtitre plates (Packard-Perkin Elmer) were sterilised in ethanol and air dried before adding 200*μ*l of molten Hoagland’s media (with additional 1% sucrose) to each well. Media containing supplemental elements was added in the same way with replicates of 24 wells (minimum) per treatment. The media was allowed to set by cooling before transferring seedlings.

Arabidopsis WS-2 *CCR2:LUC* seedlings, 7 days old, were transferred to individual wells of pre-assembled 96 well black microtitre plates, one seedling per well. The addition of 15*μ*l of 1mM potassium luciferin solution (1mM in 0.001% Triton X-100) was topically applied to each well and the plates sealed with an adhesive top seal (Perkin Elmer). The seedlings in microtitre plates were returned to entrain for a further 24 hours in the same conditions.

Assays used a TOPCount NXT liquid scintillation counter (Packard-Perkin Elmer), with plate stackers fitted with side LED banks [56].

Seedling plates were transferred to the TOPCount 24 hours after the microtitre plates were assembled, at subjective dusk. To ensure even read intervals between runs (approximately 1 hour between reads of the same plate), each assay contained 10 black seedling plates loaded alternately with clear reflector plates to allow light to pass through to the seedlings in the microtitre plates below, a 1 minute time delay and an average read over 5 seconds was used. Each experiment began with a 24–hour entrainment period in the TOPCount starting at ZT12 with 12 hours darkness. At ZT24 in the TOPCount a constant light (LL) free run period began (first missed external cue at ZT36) and measurements in LL continued for 5 days or 128 hours.

#### 2.1.5 Elements supplied

Chemicals were purchased from Sigma-Aldrich or from Thermo-Fisher Scientific and Apollo Scientific. The full list of exact chemicals used and their concentrations can be found in Tables 1–3. All elements (unless indicated) were dissolved in sterile double distilled water to make stock concentrations. The element solutions were then diluted to their final concentration in molten Hoagland’s sucrose media immediately before addition to the microtitre plate wells. For the elements described in the DEFRA/YARA/Lancrop guidelines (technical bulletin no. 10 [52]), the maximum permissible concentration was tested as well as half of the maximum concentration for the pH of the media used (5.5 < 6.0)– see Table 1. For all other chemical salts, multiple concentrations were tested. The final concentration for each (see Tables 2 and 3) was defined by the maximum concentration possible before becoming toxic to the plant. Each control and chemical salt tested contained a minimum of 24 individual Arabidopsis seedlings per experiment, and each microtitre plate contained a set of control samples.

As it is not possible to use chemical elements individually, a range of elemental compounds were used. Wherever possible, chloride salts of each element were used to make results obtained from each element as comparable as possible (because only the anion was changing). Where this was not possible, multiple combinations of compounds for the same element were tested.

### 2.2 Statistical methods

In this section, we first explain the traditional approach to statistical analysis of circadian data (FFT-NLLS) before going on to sketch the key principles behind a wavelet-based approach which, we argue, offers additional flexibility and insight.

#### 2.2.1 FFT–NLLS (Fast Fourier Transform with non–linear least squares)

Under this paradigm, a Fourier basis is chosen to represent the data, thus incurring a dependence on parameters that determine its period and shape. The parameters that define the function that best fits the data are estimated using non-linear least squares fitting. Formally, the function used to represent the data is given by:

$$\begin{array}{c} {\tilde{f}}_{FFT}\left(t\right)=\sum_{i=1}^{N}\alpha_{i}\;\text{cos}[\frac{2\pi (t-\phi_{i})}{\tau_{i}}], \end{array}$$

where *α*_*i*_ is the amplitude of each cosine, *ϕ*_*i*_ its phase, *τ*_*i*_ its period and *N* ≤ 25.

The FFT–NLLS algorithm is a two–step procedure, in which a Fast Fourier Transform (FFT) is coupled with a non–linear least squares (NLLS) fitting of cosine functions to the data [27] in the following way:

1. Remove long–term trends in the time series by fitting a linear regression model to the data and then subtracting the estimate from the original series.
2. Calculate the FFT of the transformed series.
3. Use FFT peak frequencies to sequentially (in order of descending power, up to a maximum of 25 frequencies) initialise NLLS cosine fitting (using a modified Gauss–Newton minimisation algorithm) which estimates the parameters (*τ*_*i*_, *ϕ*_*i*_, *α*_*i*_).
4. Output confidence intervals for the estimated parameters of the fitted curves.
5. Stop when the latest period estimate, ${\hat{\tau }}_{i}$ is not statistically significant or the maximum number of frequencies is reached.
6. Report all estimated significant periodicities, ${\hat{\tau }}_{1},\ldots ,{\hat{\tau }}_{l},l\leq 25$.

Under the assumption of constant period for the circadian component, its estimate is taken to be the period of the cosine component lying within a user–defined range of likely circadian periods, typically between 15 and 35 hours. If more than one cosine component is within this range, the user decides which period to select.

In Step 3, the non-linear least squares (NLLS) procedure finds parameter estimates by iteratively improving initial values via numerical search that only works well when given sensible starting values. Thus, a Fast Fourier Transform (FFT) is performed on the circadian time series to obtain good period and amplitude estimates using the data (as opposed to inputting user-defined or default values as the initial guess).

The FFT–NLLS is limited to modelling (linear combinations of) sinusoidal waveforms with constant period and does not perform well on data that are not of this type [15, 20].

#### 2.2.2 Wavelet spectral testing

A key component of our data analysis is to test whether the control and treatment groups correspond to processes that evolve in an identical spectral manner, or not. We adopt the F-testing procedure of [30], which formally corresponds to the following setup.

We denote the (time series) data by ${\left\{X_{t,T}^{\left(i\right),r_{i}}\right\}}_{t=0}^{T-1}$ with *i* = 1, 2 corresponding to one of two groups (e.g. control/ treatment) and potential replicates *r*_*i*_ = 1, …, *N*_*i*_ (i.e. *N*_*i*_ circadian traces in the *i*th group) and model it using the locally stationary wavelet (LSW) paradigm [57]:

$$\begin{array}{c} X_{t;T}=\sum_{j=1}^{J}\sum_{k\in Z}w_{j,k;T}\psi_{j,k}\left(t\right)\xi_{j,k}, \end{array}$$

where *T* = 2^*J*^ ≥ 1 is the number of sampled times, {*ξ*_*j*,*k*_} is a random orthonormal sequence of increments, {*ψ*_*j*,*k*_(*t*) = *ψ*_*j*,*k*−*t*_}_*j*,*k*_ is a set of discrete non-decimated wavelets and {*w*_*j*,*k*:*T*_} is a set of amplitudes, each of which at a wavelet scale *j* and time *k*. The increments *ξ*_*j*,*k*_ are assumed to follow a Gaussian distribution, an assumption shown to hold for circadian data [30]. The properties of the random increment sequence {*ξ*_*j*,*k*_} ensure that ${\left\{X_{t,T}^{\left(i\right),r_{i}}\right\}}_{t=0}^{T-1}$ is a zero-mean process and in practice it is customary to detrend a process with non-zero mean, which was also our approach here.

Under the LSW framework, a quantity analogous to the spectrum of a stationary process, is the evolutionary wavelet spectrum:

$$\begin{array}{c} S_{j}\left(z\right)={\left|W_{j}\left(z\right)\right|}^{2}, \end{array}$$

for each scale *j* = 1, …, *J* and rescaled time *z* = *k*/*T* ∈ (0, 1).

Assume the signals in group *i* are underpinned by a common wavelet spectrum and denote this by $S_{j}^{\left(i\right)}\left(t/T\right)$ for each group *i* = 1, 2. Therefore, we formally test:

$$\begin{array}{c} H_{0}:S_{j}^{\left(1\right)}\left(z\right)=S_{j}^{\left(2\right)}\left(z\right),\;\forall j,\;z \end{array}$$

versus the alternative $H_{A}:S_{j^{*}}^{\left(1\right)}\left(z^{*}\right)\neq S_{j^{*}}^{\left(2\right)}\left(z^{*}\right)$ for some scale j* and rescaled time z*. For simplicity, throughout the paper we refer to this test using the acronym LSW–FT.

#### 2.2.3 Wavelet–based cluster analysis

In order to facilitate the identification and characterisation of treatment effects on the plant circadian rhythm, additional to the spectral testing procedure described above, we perform cluster analysis on the chemicals listed in Table 1. Due to the demonstrated challenging features of the data, we propose to use the LSW–PCA clustering methodology of [20]. This procedure was developed to cluster inherently nonstationary rhythmic data by modelling the profiles as locally stationary wavelet processes and clustering the individual plant time-scale spectral signatures by means of a functional principal component analysis.

The LSW–PCA has been shown to be suitable for organising and understanding multiple nonstationary time series [20], and is ideally suited to the gene expression data investigated here. This analysis allows objective answers to questions such as ‘Does exposure to certain classes of soil pollutant induce a generic plant response?’.

The LSW–PCA clustering procedure can be described as follows:

1. For each plant, estimate its unknown wavelet spectrum by means of the corrected wavelet periodogram estimate (using the locits R package). Each periodogram was level smoothed by log transform, followed by translation invariant global universal thresholding and then the inverse transform was applied. For each scale of the wavelet periodogram, only levels 3 and finer were thresholded.
2. Construct a dissimilarity matrix from the profile-specific spectral information, and determine the optimal number of clusters using the methods proposed in [20].
3. Input the dissimilarity matrix and desired number of clusters obtained at Step 2 into a partition around medoids (PAM) algorithm (performed in the cluster R package).
4. Determine and characterise the plant clusters as dictated by their LUC-reponse.

## 3 Results

### 3.1 Practical considerations

For the *FFT–NLLS approach*, the Microsoft Excel software interface Biological Rhythm Analysis System (BRASS) [21] was used to perform the analysis [27] and yielded (i) period estimates (over a window of ZT36 to 120, considering only period estimates between 15 and 40 hours) and (ii) values of the relative amplitude error (RAE) for the control and treatment groups. The RAE is a value between 0 and 1 and provides information regarding the goodness of fit of the model, with a value of 0 indicating a perfect fit. Hypothesis testing (two-tailed *t*-test flagged as significant when *p* < 0.05) was then used to compare the control and treatment period estimates. Only treatment groups with an average RAE less that 0.5 were considered a statistically significant result [4, 58]. S1 Fig schematically illustrates this analysis [21]. Here, ‘period’ was defined as the time required to complete one rhythm cycle, and ‘precision’ [50] quantifies the goodness of fit of the (sinusoidal) model. A lack of precision may be associated with a non–sinusoidal rhythmic pattern (blue and pink lines in S1 Fig- bottom left panel), or with a reduction in amplitude and changing periodicity (blue line in S1 Fig- bottom right panel). Mathematically, such effects cause increases in relative amplitude error (RAE) [27].

For *wavelet representation*s, the data is often required to be of dyadic length, *T* = 2^*J*^. Therefore, as in [20, 30], our approach (for both wavelet–based methods) was to analyse a dyadic length segment of the data, with the truncation decided upon such that the selected segments contain the times during which the plants transferred into free-running conditions. The LSW–FT test [30] was used for the formal spectral comparison of nonstationary plant profiles that appear to have a nonstationary behaviour. As illustrated in the S2 Fig, the wavelet testing results can be visualised in a ‘barcode plot’ [30]. Here, a black line indicates a statistically significant (*p* < 0.05) difference in the spectral behaviour of the treatment group (relative to the control), at a given time–frequency location.

The *multiple-hypothesis testing* aspect was dealt with by implementing a False Discovery Rate (FDR) approach and Bonferroni correction.

### 3.2 Data analysis and findings

For each chemical treatment group, Tables 1–3 report (i) the difference in the mean period estimates for the appropriate control and treatment group, (ii) the mean relative amplitude error (RAE) and (iii) the number of rejections (as a percentage in brackets) for the LSW–FT with FDR at the 5% significance level.

In Section 3.2.1 we show that excess external chemicals have varying effects on plant circadian rhythms, with Fig 1 displaying examples of statistically significant period shortening and period lengthening (*p* < 0.05) and clear circadian disruption not captured by FFT–NLLS. Changes in period are discussed in detail in Section 3.3.1 while other effects such as the induced lack of precision, are discussed in Section 3.3.2. In Section 3.4 we discern whether the effect of a certain element on the circadian clock is dose–dependent, and whether different chemical treatments have distinct effects on the clock as opposed to a generic excess chemical response.

#### 3.2.1 Excess external chemicals have varying effects on plant circadian rhythms

**Fig 2:**
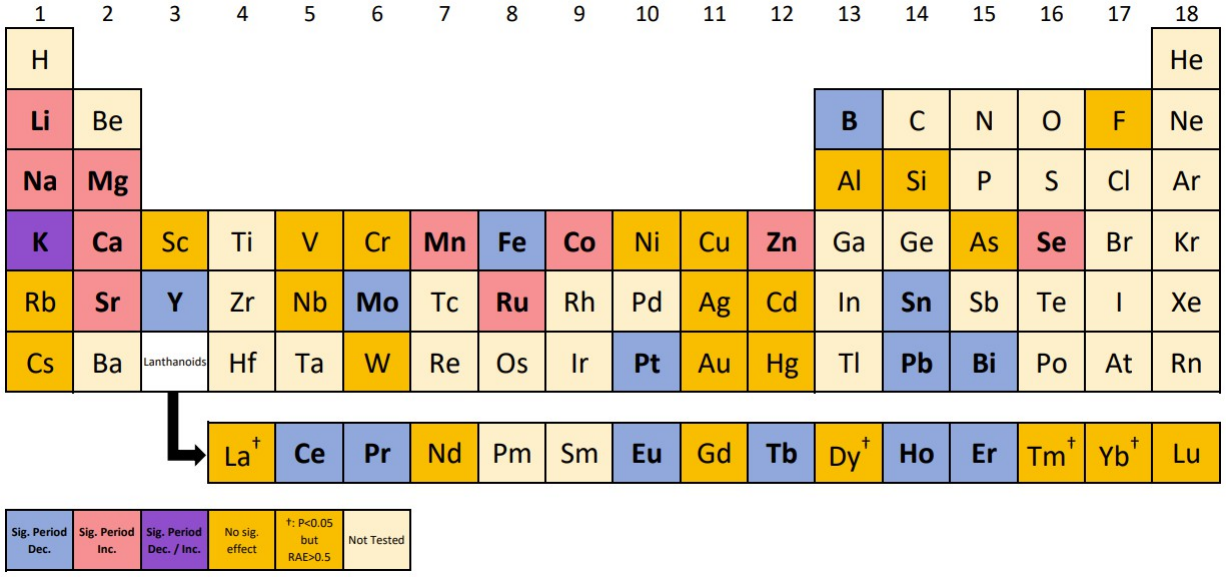
The effect on period using FFT-NLLS methods. An element (see Tables 1–3) is defined as having a significant effect (and denoted in **bold**) if at least one corresponding chemical treatment gave a significant result (*p* < 0.05 and *RAE* < 0.5). Colours denote the type of induced change (see key).

Using FFT–NLLS, 29 out of the 76 chemical treatments tested were found to induce a statistically significant change in period (*p* < 0.05, *RAE* < 0.5, see Tables 1–3) and 25 out of 49 elements investigated induced at least one statistically significant change in period (*p* < 0.05, *RAE* < 0.5, summarised in Fig 2). Five chemical treatments (Na_2_MoO_4_ (Max), LaCl_3_, DyCl_3_, TmCl_3_ and YbCl_3_) caused a significant change in period (*p* < 0.05) but also induced a lack of precision (*RAE* > 0.5) which indicates that simply reporting a period change is not credible for these treatments.

**Fig 3:**
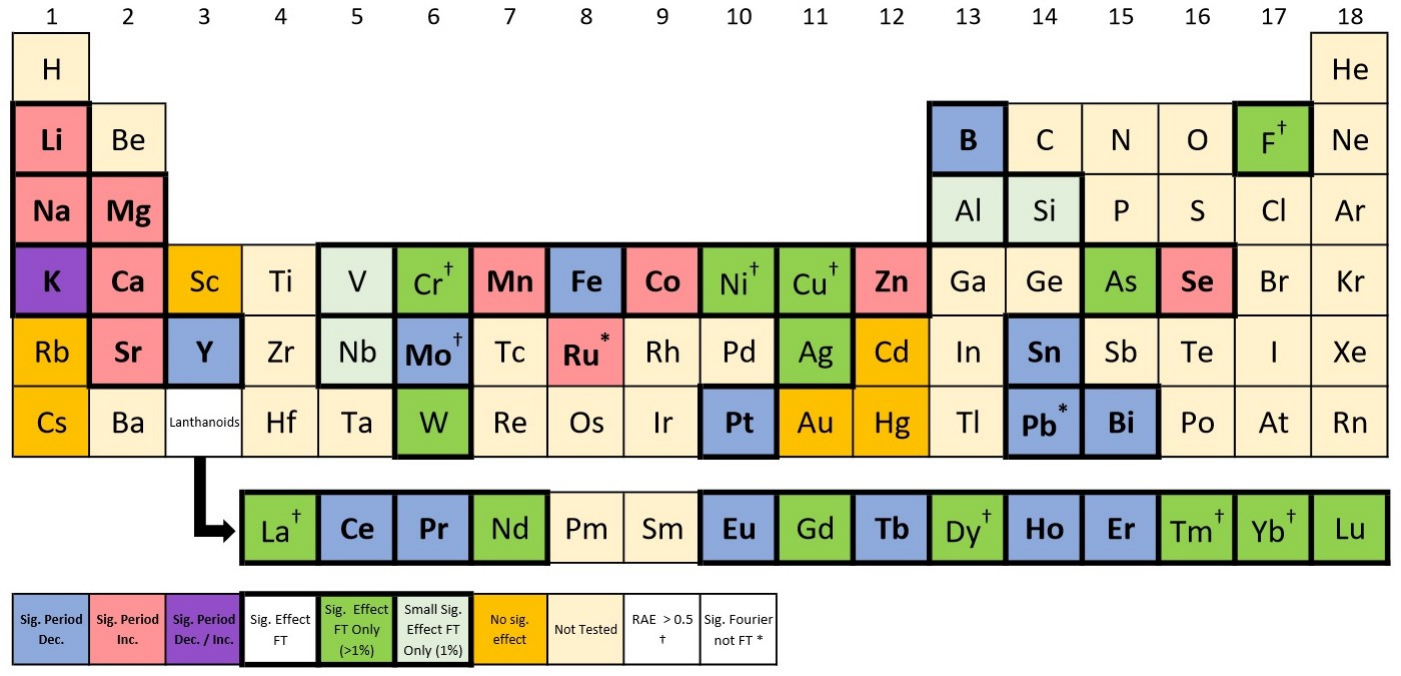
Combined output of FFT–NLLS and LSW–FT methods allows further insight into the impact of excess elements. For each method, an element (see Tables 1–3) is defined as having a significant effect if at least one corresponding chemical treatment gave a significant result (a statistically significant result which also follows the specific method’s usability criteria). Colours/ **bold font**/ bold outline denote the type of induced change (see key).

Using the LSW–FT with false discovery rate (FDR) to adjust for the multiple hypothesis-testing, we found that 57 out of the 76 chemical treatments tested induced a statistically significant change in the Arabidopsis clock (*p* < 0.05, see Tables 1–3) and 42 out of the 49 elements investigated induced (at least one) statistically significant change in precision (*p* < 0.05, summarised in Fig 3). The five chemical treatments identified by FFT–NLLS to cause a significant change in period (*p* < 0.05) and to also induce a lack of precision (*RAE* > 0.5) have also been found by LSW–FT to induce a significant effect (*p* < 0.05) on the circadian clock.

Across the FFT–NLLS and the LSW–FT methodologies, 59 out of the 76 chemical treatments tested were found to induce a statistically significant change in the Arabidopsis clock and 43 out of 49 elements investigated induced (at least one) statistically significant change (summarised in Tables 1–3 and Fig 3, respectively). In particular, for six elements (Scandium (Sc)– both compounds, Rubidium (Rb), Cadmium (Cd)– both concentrations of both compounds, Caesium (Cs), Gold (Au) and Mercury (Hg)– both concentrations) there was not sufficient evidence to deem that a significant effect occurs in the circadian clock.

With the exception of Ruthenium (Ru) and Lead (Half), every chemical treatment that induced a statistically significant change in period (*p* < 0.05, using FFT–NLLS) was also found to cause a statistically significant change in precision (*p* < 0.05, using LSW–FT). This is denoted by the * symbol in Fig 3.

When using the LSW–FT testing, practitioners can be (cautiously) informed by the number of rejections identified using the wavelet–spectral testing methodology (as a dissimilarity measure), with larger values indicating a greater difference between the control and chemical treatment groups [30]. For our dataset, a 1% percentage of rejections is classified as a ‘small’ change in the Arabidopsis clock, with larger values of percentage rejections indicating a greater departure from the control behaviour. Such a ‘small’ change was identified in four elements (Aluminium (Al), Silicon (Si), Vanadium (V) and Niobium (Nb), see Fig 3). This is in agreement with the FFT–NLLS analysis which found changes in period that were not statistically significant (*p* > 0.05).

We also used the LSW–PCA clustering algorithm for the spectral signatures corresponding to the treatment plant profiles listed in Table 1. Two principal components were retained and all methods (described in Section 2.2.3) indicated that the data should be clustered into two groups, thus supporting the purpose of this analysis. The results are detailed in Table 4 and the two identified groups correspond to whether or not a chemical effect on the clock is detected.

**Table 4:** Results of the cluster analysis reported in Section 3.2.1.

Treatment Group	Number of Plants in Cluster 1	Number of Plants in Cluster 2
Control 1	17	7
Copper (Max)†‡	24	0
Selenium (Max)*†‡	24	0
Control 2	14	10
Lead (Half)*	19	5
Mercury (Max)	20	4
Control 3	17	7
Lead (Max)†‡	24	0
Control 4	15	9
Selenium (Half)*†‡	24	0
Cadmium (Half)	20	4
Control 5	10	14
Zinc (Max)*†‡	24	0
Control 6	16	8
Molybdenum (Max)†‡	24	0
Molybdenum (Half)*†‡	24	0
Control 7	16	8
Arsenic (Max)†‡	24	0
Arsenic (Half)†‡	24	0

The chemical treatments (and their respective controls) were clustered into two groups using the LSW–PCA clustering methodology. Note: There are 24 plants in each treatment group;

* indicates a significant period increase/ decrease using FFT–NLLS;

^†^ indicates a significant change in spectral behaviour using wavelet spectral testing;

^‡^ indicates a treatment with 0 plants in Cluster 2.

Of the investigated chemical treatments, five (Zinc (Max), Selenium (both), Molybdenum (Half) and Lead (Half)) had a statistically significant effect according to the FFT–NLLS analysis and nine (Copper (Max), Zinc (Max), Arsenic (both), Selenium (both), Molybdenum (both) and Lead (Max)) had a statistically significant effect according to the LSW–FT testing. Both methods found that Zinc (Max), Selenium (both) and Molybdenum (Half) have a significant effect on the Arabidopsis clock. The LSW–FT test additionally found that Copper (Max), Arsenic (both), Molybdenum (Max) and Lead (Max) had a significant effect. However, while the testing methods disagree on whether Lead (Half) has a significant effect, the clustering results help nuance these findings.

On examining Table 4, we note the LSW–PCA clustering method has clustered the behaviour of the data into the following two groups: Cluster 2 identifies similar behaviour of plants in the control groups and the Lead (Half), Mercury (Max) and Cadmium (Half) treatment groups, while Cluster 1 contains all 24 plants in the remaining chemical treatment groups. These results suggest that the plants in the Lead (Half), Mercury (Max) and Cadmium (Half) treatment groups seemed to display similar behaviour to the control groups, indicating that these chemicals have no effect on the circadian clock. This conclusion is fully supported by the LSW–FT analysis which found no rejections of the null hypothesis of spectral equality for these chemicals, and partially supported by the FFT–NLLS analysis which found no significant change in period for Mercury (Max) and Cadmium (Half) treatment groups, but did indicate a significant change in period for the Lead (Half) treatment group. We thus conceptualise Cluster 2 as essentially ‘No Change’ and Cluster 1 as ‘Change’.

### 3.3 Excess external chemicals affect various rhythmic parameters

#### 3.3.1 Excess external chemicals affect period

*Chemical treatments induce a decrease in period*. Using the FFT–NLLS methodology, 15 out of the 76 chemical treatments tested induced a statistically significant decrease in period (*p* < 0.05, *RAE* < 0.5, see Tables 1–3) and 14 out of 49 elements investigated induced (at least one) statistically significant (*p* < 0.05, *RAE* < 0.5) decrease only in period, summarised in Fig 2 by the blue–coded elements. For example Boron (B) excess, significantly (*p* < 0.05) shortened the circadian period of *CCR2:LUC* activity by almost 2 hours on average when compared with B-replete controls (see Panel (a) of Fig 1). The five chemical treatments discussed in Section 3.2.1 (Na_2_MoO_4_ (Max), LaCl_3_, DyCl_3_, TmCl_3_ and YbCl_3_) found to cause a significant change in period (*p* < 0.05) and to induce a lack of precision (*RAE* > 0.5), all decreased the period.

*Chemical treatments induce an increase in period*. Using the FFT–NLLS methods, 14 out of the 76 chemical treatments tested induced a statistically significant increase in period (*p* < 0.05, *RAE* < 0.5, see Tables 1–3) and 10 out of 49 elements investigated induced (at least one) statistically significant (*p* < 0.05, *RAE* < 0.5) increase (only) in period (summarised in Fig 2 by the red–coded elements). For example, Magnesium (Mg) excess significantly (*p* < 0.05) lengthened the circadian period of *CCR2:LUC* activity by approximately 2 hours on average when compared with Mg-replete controls (see panel (b) of Fig 1).

*Excess potassium both increases and decreases period depending on compound*. Depending on the chemical compound, Potassium (K) excess was shown to both significantly (*p* < 0.05) increase and decrease the circadian period of *CCR2:LUC* activity (see Table 2). Both KCl and KBr (with K at a concentration of 100*mM*) significantly (*p* < 0.05) lengthened the circadian period by approximately 90 minutes on average when compared with K-replete controls. However, KI (with K at a concentration of 100*mM*) significantly (*p* < 0.05) shortened the circadian period by approximately 90 minutes on average.

#### 3.3.2 Excess external chemicals induce a lack of precision

Mathematical analysis confirmed that plant rhythms subjected to (one of) nine of the elements investigated (Fluorine (F), Chromium (Cr), Nickel (Ni), Copper (Cu), Molybdenum (Mo), Lanthanum (La), Dysprosium (Dy), Thulium (Tm) and Ytterbium (Yb)) were significantly less robust than displayed by the control.

Using the FFT–NLLS analysis, 11 out of the 76 chemical treatments tested induced a statistically significant increase in RAE (*p* < 0.05): the RAE exceeded the 0.5 threshold for *CCR2* rhythm in the treatment group, whereas it was approximately 0.2 in the control (see Tables 1–3). Out of the 49 elements investigated, 9 induced (at least one) statistically significant increase in RAE (indicated by the † symbol in Fig 2).

All of the above chemicals were detected to induce a statistically significant change in the precision of the Arabidopsis clock (*p* < 0.05, see Tables 1–3) by the wavelet testing methodology LSW–FT.

**Fig 4:**
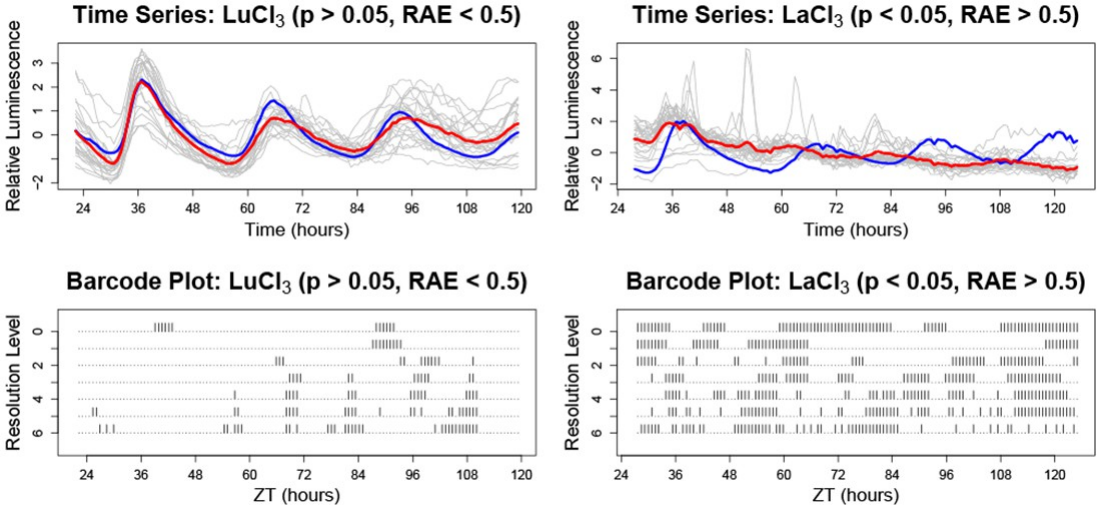
Detecting spectral differences using wavelet spectral testing. Examples (Fig 1 panels (c) and (d)) showing clear circadian disruption not captured by FFT–NLLS (*p* > 0.05 or *RAE* > 0.05), but captured by LSW–FT. Top panels: individuals in the treatment group (grey) are shown along with the treatment group average (red) and the control group average (blue). Each time series has been standardised to have mean zero. Lower panels: Barcode plots indicating the time–scale locations of significant spectral differences (LSW–FT with FDR, *p* < 0.05) between the control and treatment group.

For example, Lanthanum (La) regulates clock precision under LL and the effects can be observed throughout the experiment (see panel (d) of Fig 1). Mathematical analysis confirmed that, throughout the experiment, the circadian rhythms were significantly less robust than observed in the control: the ‘barcode plot’ (see the bottom–left panel of Fig 4) indicates that significant differences (between the treatment and control groups) are present at all timepoints, throughout LL free-run. Lack of precision was associated with a loss of rhythmicity (i.e. increased amplitude of *CCR2* oscillations (ZT24–ZT60), followed by decreased amplitude (ZT60–ZT120)) and an increased RAE (Table 3). Lanthanum (La) is therefore important for the maintenance and precision of circadian rhythms, especially under LL.

#### 3.3.3 Insights from wavelet analyses where FFT–NLLS fails

Using the LSW–FT methodology, we found that 51 of the 76 chemical treatments tested and 14 of 49 elements investgated induced a stronger change in the Arabidopsis clock (LSW–FT with FDR, *p* < 0.05), but crucially did not display a statistically significant change in period (FFT–NLLS, *p* < 0.05, see Tables 1–3). This is represented in Fig 3 by the (darker) green–coded elements. As discussed in Section 3.3.2, many of these chemicals induced a lack of precision, indicated by an RAE above the 0.5 threshold, which explains why traditional period estimation techniques would not identify this change. However, 10 chemical treatments (NaF (Fluorine– Half); KAsO_4_(Arsenic–both concentrations); AgNO_3_ (Silver); Na_2_WO_4_ (Tungsten); NdCl_3_ (Neodymium); GdCl_3_ and (CF_3_SO_3_)_3_Gd (Gadolinium); DyF_3_ (Dysprosium); LuCl_3_ (Lutetium)) had an average RAE less than 0.5.

For example, Lutetium (Lu) excess significantly affects the rhythmicity of *CCR2:LUC* activity when compared with Lu-replete controls (LSW–FT with FDR, *p* < 0.05), however they do appear to preserve rhythmicity (see panel (c) of Fig 1). This is confirmed mathematically with an average RAE of 0.25 (Table 3). However, this change manifests after ZT84 and it can be seen in the ‘barcode plot’ in the bottom–right panel of Fig 4. This delayed response may explain why this change of period is not detected by the traditional period estimation techniques.

### 3.4 Identifying chemicals with similar effects on the clock

#### 3.4.1 Similarity in difference

The wavelet–based methods used for our analysis crucially facilitate objective ways to capture similarities in the effects of specific chemicals on the circadian clock. This allows us to address questions such as ‘Does exposure to different elements in the periodic table produce a generic type of reaction in plants?’ and, if this is not the case, then ‘Which elements induce similar kinds of reactions in plants?’

In a bid to answer these questions, we also applied the LSW–PCA clustering methodology to one of the individual microtitre plates (labelled 0953) within the chemical dataset corresponding to chemical treatments with Lead and Chromium (Table 1). Note that while in Section 3.2.1 we clustered data arising from different microtitre plates, now we perform the wavelet–based analysis on treatment (excluding control) profiles from the same plate [20, 30].

**Fig 5:**
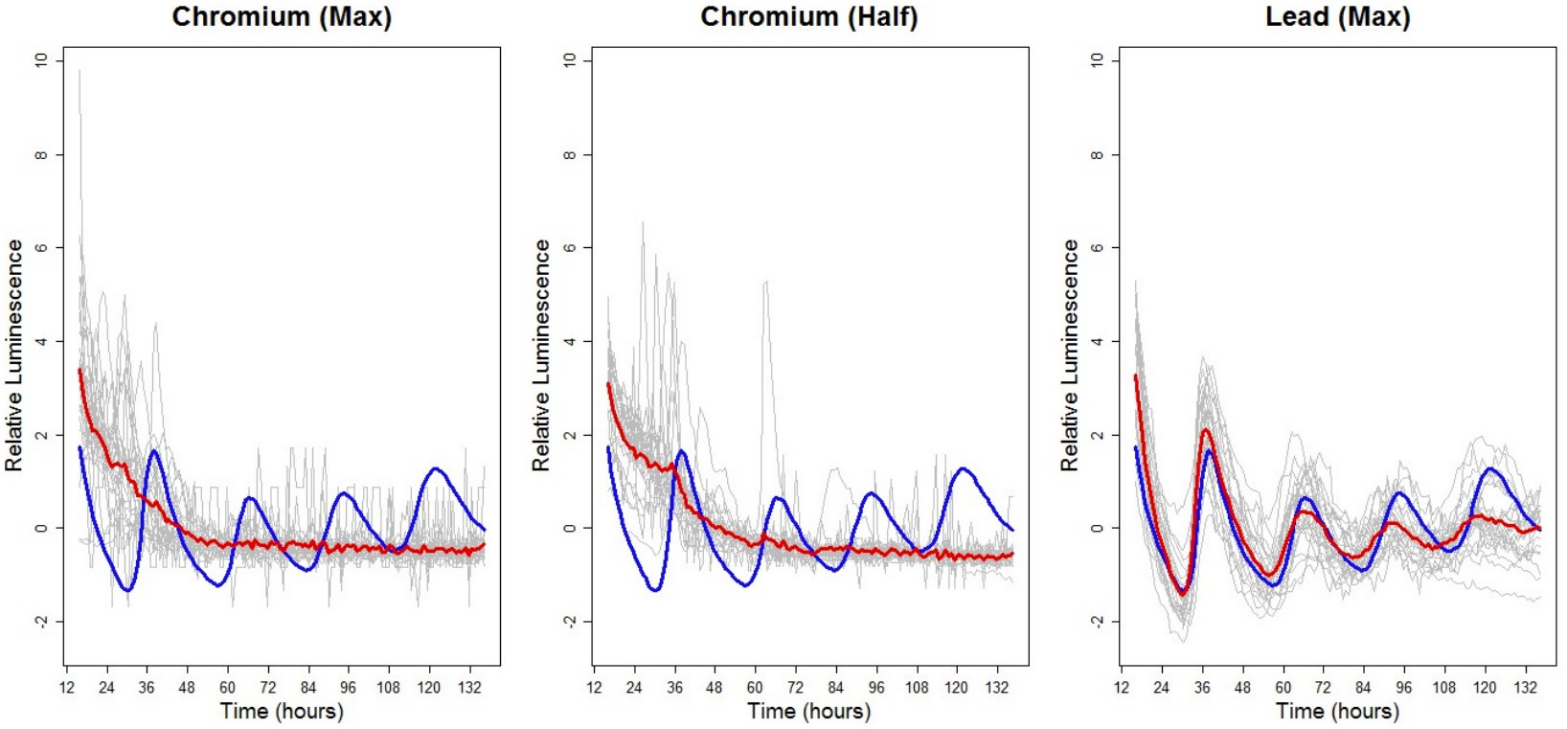
Examples of variability within and between treatments: Luminescence profiles over time for *A. thaliana* plants exposed to (different concentrations of) Chromium and Lead (see Table 1 for more details). Each panel: individuals in the treatment group (grey) are shown along with the treatment group average (red) and the control group average (blue). Each time series has been standardised to have mean zero.

The LSW–PCA clustering method was used to analyse the following three chemical treatments: Chromium (Half and Max concentrations) and Lead (Max). Fig 5 displays the individual time series for these chemical treatments. On examining the screeplot and for ease of interpretation, we retained two principal components to cluster this data and all methods of [20] indicated that we should cluster the data into two groups. The LSW–PCA clustering method yielded the results detailed in Table 5.

**Table 5:** Results of the cluster analysis reported in Section 3.4.1.

Number of plants	Chromium (Max)	Chromium (Half)	Lead (Max)	Total
Cluster 1	**24**	**24**	9	57
Cluster 2	0	0	**15**	15
Total	24	24	24	72

The chemical treatments (Chromium (Half and Max concentrations) and Lead (Max)) were clustered into two groups using the LSW–PCA clustering methodology. Note: The modal cluster for each treatment group is highlighted in bold.

On examining Table 5, the following two behaviour groups arise: Cluster 1 identifies similar behaviour of plants in both Chromium treatment groups (conceptualised as essentially ‘Chromium’) and Cluster 2 is the modal cluster of the Lead (Max) treatment group (conceptualised as ‘Lead (Max)’). These results are in agreement with Fig 5 which provided visual evidence that the plants in both Chromium treatment groups displayed similar behaviour, while the Lead (Max) group displayed average behaviour which is distinct from the Chromium groups (and from the control group).

The LSW–FT methodology (see Section 2.2.3) was implemented to test for differences between both Chromium treatment groups and the Lead (Max) group and the results appear in Table 6, thus confirming the conclusions of the LSW–PCA clustering that although all three chemicals have an effect on the Arabidopsis circadian clock (Table 1), they do not induce the same effect. This is also reflected in the higher number of percentage rejections when comparing the Lead (Max) group with the Chromium groups (approximately 60%), than the Chromium groups with each other (29%). This suggests that the chemicals do not simply induce a generic chemical stress response, and induce a chemical–specific response.

**Table 6:** Variability within and between treatments: Results of the LSW–FT (FDR) analysis reported in Section 3.4.1.

Chemical Treatment Group 1	Chemical Treatment Group 2	Rejections FT (FDR)
Chromium (Max)	Chromium (Half)	264 (29%)
Chromium (Max)	Lead (Max)	553 (62%)
Chromium (Half)	Lead (Max)	533 (59%)
Chromium (both)	Lead (Max)	576 (64%)

For each pair of the chemical treatments (Chromium (Half and Max concentrations) and Lead (Max)), the number of rejections (as a percentage in brackets) for the LSW–FT with FDR (at the 5% significance level) is reported.

#### 3.4.2 Dose–dependent effect on the clock precision

Having established that albeit Chromium (both concentrations) and Lead (Max) have an effect on the circadian clock, both concentrations of Chromium appear to have a similar effect but distinct to the effect of Lead (Max), here we test whether the effect of Chromium is dependent on dose level.

On examining Table 6, we note that there are still a large number of rejections of the null hypotheses of spectral equality between the two chromium treatment groups, hence although the Chromium treatment groups have a higher degree of similarity than the Lead (Max) group, they are still significantly different.

The wavelet–based hypothesis tests thus indicate that Chromium induces a lack of precision in the Arabidopsis clock, in a dose–dependent manner.

## 4 Discussion

There are many possible mechanisms for the causes for, and consequences of, the effects we report here. Cations can be nutritional and/or toxic, in that a given cation may interfere with normal cellular responses, alter signalling, or act to alter the osmotic potential of the soil. It is interesting that many anions also have circadian performance consequences. Many effects will be synergistic; such a response is well reported for the interaction of iron and zinc [38, 59–61].

Magnesium (Mg) deficiency dampens the amplitude of circadian clock genes [19, 37] and increases the period of *pCCA1:LUC* oscillation [19]. In Section 3, our analysis indicated that excess Mg has a significant effect on the Arabidopsis circadian clock. The FFT–NLLLS analysis found that excess magnesium also increases period of *pCCR2:LUC* oscillation. This supports the assertion in [19] that magnesium maintains the length of circadian period in Arabidopsis.

Iron (Fe) deficiency increases the period of key Arabidopsis circadian clock components [38, 59, 62]. In Section 3, our analysis indicated that excess Fe has an effect on the Arabidopsis circadian clock. The FFT–NLLLS analysis found that excess magnesium also decreases period of *pCCR2:LUC* oscillation. These findings support others’ findings that sufficient Fe supply is required to support timekeeping in plants [38, 59, 62].

Copper (Cu) deficiency has also been shown [4] to increase the amplitude of two of the main components of the Arabidopsis central oscillator- CIRCADIAN CLOCK ASSOCIATED1 (CCA1) and LATE ELONGATED HYPOCOTYL (LHY). However, it is less well understood how Cu affects period with some studies concluding that period remains mostly unaffected [63] and others suggesting a dynamic change in period [20]. One reason for the conflicting conclusions it the strengths and limitations of the period estimation techniques employed [15, 30]. Our analysis in Section 3 supports these conclusions. The FFT–NLLLS analysis found that excess Cu had no statistically significant effect on the period of *pCCR2:LUC* oscillation, supporting the results [63]. However, this analysis also found an RAE value over 0.5, indicating a lack of precision. This suggests that Cu has an impact on the circadian clock, but these changes may not be limited to a simple change in period. Therefore, the wavelet–based methods may be more appropriate for this dataset. In fact, the wavelet spectral testing found that Cu had a significant impact on the circadian clock. These findings suggest that sufficient Cu supply is required to support proper timekeeping in plants.

A number of studies have investigated the interaction between nutritional status and circadian rhythms. We have found that the exogenous supply of many metals have distinct action clock rhythms. Current industrial use of lithium, sodium, calcium, cobalt, zinc, manganese and potassium are other metals that when present in the soil would be predicted to alter the architecture of rhythmic parameters.

Regardless of the mechanisms of action for the huge array of effects we have seen, there are clear implications of our work on soil–plant interactions on circadian clock function. In the future, it will be similarly interesting to see the impact ion deficiencies have on clock performance, as mineral deplete soil is increasingly becoming a global issue. Here we note the role of supplementing macro- and micro-nutrients in fertiliser and the pervasive effects of these in intensive farming, and heavy metal contamination. The effects of these on on clock-controlled outputs need to be explored, such as seasonal control of flowering time and autumn leaf senescence.

The results presented here describe and synthesise what is, to our knowledge, the broadest set of data relating to the impact of soil pollutants on plant circadian rhythms within a standardised experimental setting. The results are, however, necessarily constrained; other pollutants could be tested in future studies, and the responses of alternative plant species may be investigated. This work shows how such experimental data can be presented within a general framework for data analysis, and allows for further objective comparisons to be made as more data are generated.

The overall picture which emerges from this study is unsurprisingly complex. Different soil pollutants can increase, or decrease, the period of the circadian clock, and can also disrupt the rhythm to an extent that data analysis based purely on periodic metrics is rendered ineffective. In such cases, we have shown that wavelets offer a practical and tractable additional method for time series analysis. The ability to cluster the responses to soil pollutants using these more subtle methods has been shown to reveal new signals in the data; in addition to changes in period, phenomena such as loss of precision and dose-dependence emerge.

Our results show the added value of wavelet methods, including hypothesis testing and clustering, for biological time series data, demonstrating that they can distinguish biologically relevant differences in responses to a wide range of environmental factors. Crucially, the removal of any need for statistical stationarity in the dataset is avoided, and indeed this generalisation allows for the more sophisticated comparisons outlined above. We have already shown that the general approaches explained here can be used for other circadian applications [20, 30], and the challenge remains to apply the framework to other experimental time series data.

## Supporting information

S1 Fig The defined rhythmic parameters: periodicity, phase, amplitude and clock precision (taken from [20] based on an image from [4]). Zeitgeber time in text is ‘ZT’.
(TIF)

S2 Fig Detecting spectral differences using wavelet spectral testing. Barcode plots corresponding to Fig 1(a) and 1(b) showing where we can see significant differences using LSW–FT.
(TIF)

S1 Dataset (ZIP)
