# Supplementary figures and images for: Multiple metals influence distinct properties of the Arabidopsis circadian clock
Source: PLoS One. 2022 Apr 5;17(4):e0258374. doi: 10.1371/journal.pone.0258374 (PMC8982871; doi:10.1371/journal.pone.0258374)

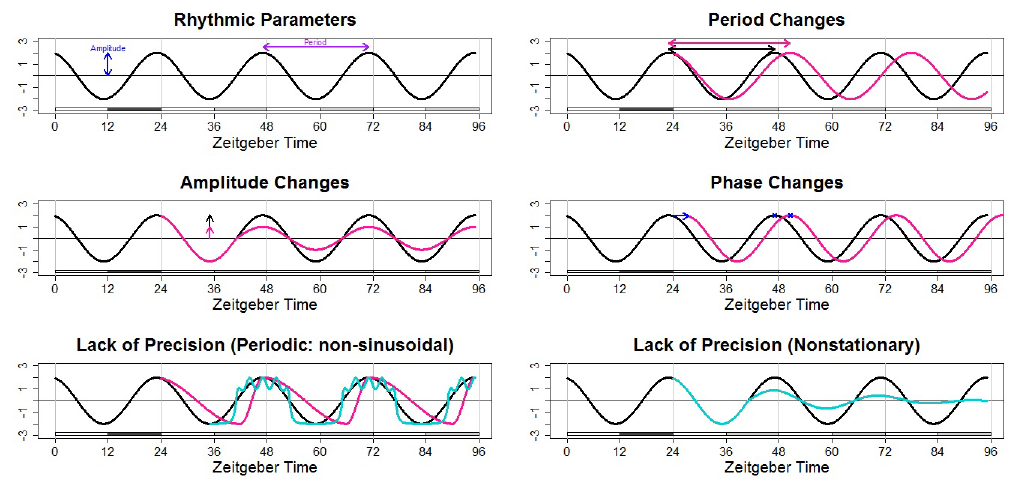

Supplement: S1 Fig — Zeitgeber time in text is ‘ZT’. (TIF) [file pone.0258374.s001.tif]

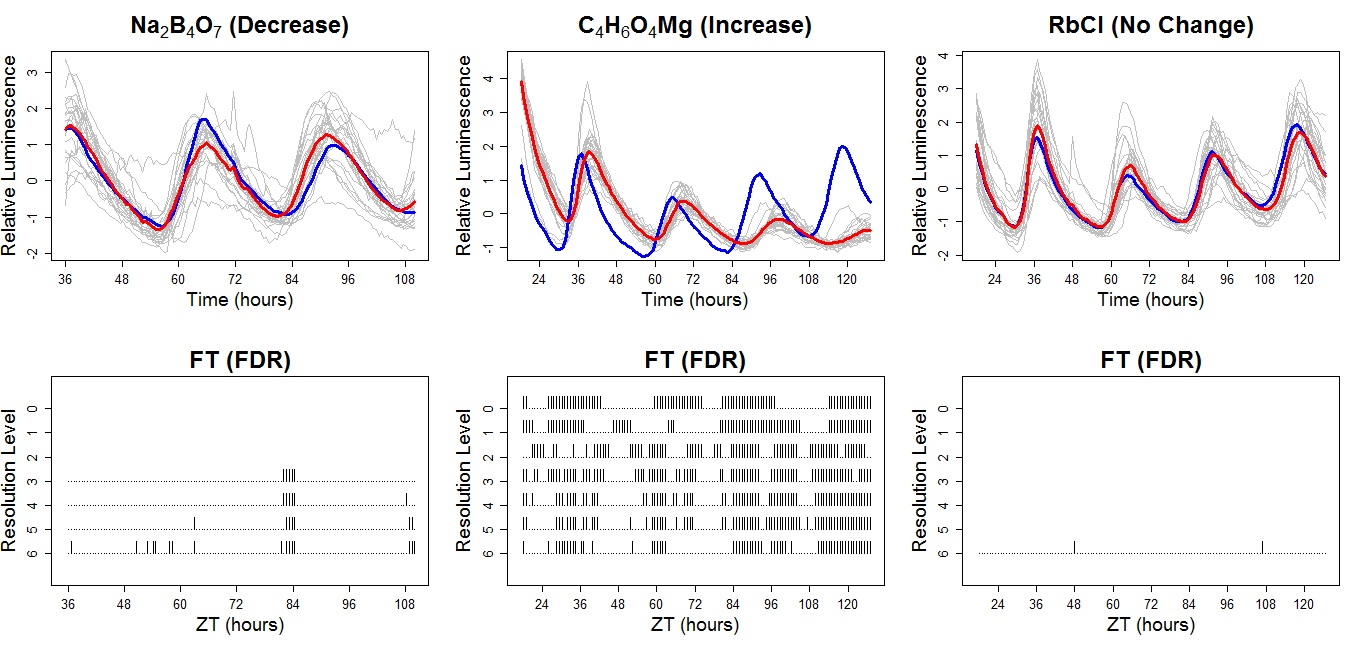

Supplement: S2 Fig — Barcode plots corresponding to Fig 1(a) and 1(b) showing where we can see significant differences using LSW–FT. (TIF) [file pone.0258374.s002.tif]
