# Supplementary material for: Multiple metals influence distinct properties of the Arabidopsis circadian clock
Source: PLoS One. 2022 Apr 5;17(4):e0258374. doi: 10.1371/journal.pone.0258374 (PMC8982871; doi:10.1371/journal.pone.0258374)
Supplement: S1 Dataset — (ZIP) [file pone.0258374.s003.zip › Supporting Information (Dataset)/Read_Me.pdf]

Handbook: Primary Data Files

Jessica K. Hargreaves[1*◕], Rachael J. Oakenfull[2◕], Amanda M. Davis[2], Freya Pullen[2], Marina I. Knight[1], Jon W. Pitchford[1,2], Seth J. Davis[2*,3]

1 Department of Mathematics, University of York, York, UK
2 Department of Biology, University of York, York, UK
3 State Key Laboratory of Crop Stress Biology, School of Life Sciences, Henan University, 475004 Kaifeng, China

* = corresponding authors: jessica.hargreaves@york.ac.uk (correspondance for mathematics); seth.davis@york.ac.uk (correspondance for circadian measurements).
◕ = joint first authors

1 Overview

This document provides a guide to the primary data files that contain the data originally analysed in the manuscript, "**Multiple metals influence distinct properties of the Arabidopsis circadian clock**" (orignally published in PLOS ONE in 2022). The authors of this manuscript are as listed above.

2 A guide to this dataset

This document provides a guide to this dataset. This dataset consists of four files:

- Results Table
- Table 1
- Table 2
- Table 3.

2.1 Results Table

This file collates the results contained in Tables 1, 2 and 3 in the original manuscript.

2.2 Table 1, Table 2 and Table 3

Each of these files follow the same general format and correspond to the equivalently named table in the original manuscript. The results contained in the corresponding table in the original manuscript are displayed on the first tab (along with some additional details).

Column A ("Label") corresponds to a tab within the spreadsheet. In this tab, the raw (TOPCount) time series data for each plant can be found. The time series for each plant is displayed in columns and each row corresponds to one time point (the ZT Time is reported in column C). We report the (primary) time series data for both the chemical treatment group and the corresponding control group (containing growth medium only)– delineated by the "Label" in row 7.

2.3 Further details

For further details (including the experimental procedures that gave rise to this dataset), please see the original manuscript.
